# Barriers to effective sample management in viral load testing in Machakos County, Kenya: a convergent parallel mixed-method study

**DOI:** 10.11604/pamj.2025.51.26.46991

**Published:** 2025-05-29

**Authors:** Christine Mutewa Kathinzi, Peter Kariuki Njenga, Joseph Kiplangat Mutai

**Affiliations:** 1 Jomo Kenyatta University of Agriculture and Technology, School of Public Health, Department of Environmental Health and Disease Control, Nairobi, Kenya,; 2 Jomo Kenyatta University of Agriculture and Technology, School of Biological Sciences, Nairobi, Kenya,; 3 Kenya Medical Research Institute, Centre for Public Health Research Department, Nairobi, Kenya

**Keywords:** Viral load, sample collection, barriers of sample management, laboratory efficiency, HIV viral load monitoring

## Abstract

**Introduction:**

viral load (VL) monitoring is a critical component of HIV management, yet systemic and logistical barriers compromise the quality and reliability of VL sample management in many low- and middle-income countries. In Machakos County, Kenya, these challenges persist, contributing to a relatively low viral suppression rate of 81% and achievement of UNAIDS 95-95-95 strategy. This study determined barriers to effective VL sample management in Machakos County, Kenya, with a focus on equipment maintenance, human resource capacity, and supply chain performance across public and private facilities.

**Methods:**

a convergent parallel mixed-methods design was employed across 71 health facilities (61 public, 10 private) served by four VL hubs: Machakos Level 5, Matuu Level 4, Athi River Level 4, and Kangundo Level 4 hospitals. Quantitative data was collected from 205 healthcare workers using structured questionnaires. Descriptive statistics, Fisher’s Exact Test, and Odds Ratios (OR) with 95% Confidence Intervals (CI) assessed associations between barriers and sample management outcomes. Qualitative data was obtained through 38 key informant interviews with clinicians from Comprehensive Care Clinics and Maternal and Child Health units (public n=32, private n=6). Transcripts were thematically analyzed using Braun and Clarke’s framework, and a word cloud visualized common terms. Findings were triangulated for contextual depth. Ethical approval was obtained from the Kenya Medical Research Institute- Scientific Ethics Review Unit, with clearance from the Machakos County Department of Health. Informed consent was obtained and confidentiality were strictly maintained.

**Results:**

among the VL hubs assessed, 88.8% reported having a designated VL focal person, and 73.2% indicated that couriers had received some form of training. Despite these structural provisions, critical technical gaps persisted. Only 6.8% of facilities had calibrated centrifuges, 2.4% conducted preventive maintenance, and 2.4% calibrated their temperature monitoring devices-pointing to widespread neglect of equipment quality assurance protocols. Further, 58.5% of facilities reported incidents of sample freezing, often attributable to inconsistent cold chain practices. These deficits were not isolated but interrelated, collectively undermining the integrity of VL sample handling. Bivariate analysis revealed statistically significant associations between effective sample management and calibrated temperature devices (OR: 3.4; 95% CI: 1.6-7.0; p = 0.01), absence of sample freezing (OR: 2.8; 95% CI: 1.3-6.2; p= 0.03), and trained couriers (OR: 1.5; 95% CI: 0.9-2.7; p= 0.06), accentuating the importance of technical reliability alongside human resource readiness. The VL hubs with preventive maintenance showed significantly higher odds of effective sample management (OR: 4.5; 95% CI: 2.0-10.1; p< 0.001). Only 5.4% of facilities reported consistent availability of VL collection materials. Qualitative insights highlighted sporadic equipment servicing, recurrent stockouts, and poor coordination between facility-level operations and county logistics.

**Conclusion:**

persistent systemic barriers-such as inadequate equipment maintenance and fragmented supply chain management-undermine the effectiveness of VL sample management in Machakos County. Strengthening technical quality protocols, institutionalizing regular equipment maintenance, and enhancing supply chain coordination through the National AIDS and STI Control Program (NASCOP) and county health departments are critical to improving diagnostic reliability and advancing Kenya’s HIV viral suppression targets.

## Introduction

Viral Load testing plays a critical role in HIV/AIDS management by monitoring treatment efficacy, detecting virologic failure, and guiding antiretroviral therapy (ART) adjustments. However, effective sample management remains a significant challenge, particularly in resource-limited settings such as Kenya. The integrity of VL samples is contingent upon a well-functioning healthcare infrastructure, adequate human resource capacity, and reliable equipment maintenance. Despite ongoing efforts to enhance laboratory systems, multiple barriers persist, adversely impacting the quality of VL monitoring [1].

Empirical evidence affirms the importance of equipment maintenance in ensuring the integrity of VL samples. The study by Afzal *et al*. highlights that frequent equipment malfunctions, reagent stockouts, and inadequate calibration procedures contribute to erroneous VL measurements [2]. The reliability of diagnostic equipment is crucial for ensuring accurate VL quantification, and poor maintenance practices increase the risk of data variability, leading to suboptimal clinical decision-making. Additionally, laboratories in sub-Saharan Africa often experience extended downtimes due to delays in obtaining spare parts and limited access to biomedical engineers trained in equipment servicing. These disruptions not only compromise sample processing timelines but also result in increased turnaround times for test results, thereby delaying critical interventions [3].

Moreover, delays resulting from equipment malfunctions directly impact VL sample integrity by prolonging storage periods, which can lead to sample degradation, hemolysis, or microbial contamination, all of which compromise diagnostic accuracy. Studies have shown that the quality of VL results deteriorates with prolonged storage, particularly when samples are not stored under optimal conditions. For instance, Omooja *et al*. found that delayed processing of dried blood spot (DBS) samples led to lower VL detection rates [4], while fresh plasma samples consistently provided accurate readings [4,5]. Such evidence highlights the importance of maintaining an efficient sample management system to ensure reliable VL results.

The role of human resources in VL sample handling is equally pivotal. Studies indicate that laboratory personnel shortages, inadequate training, and high staff turnover rates adversely affect the efficiency of VL testing programs [6]. The complexity of VL sample collection, storage, and transport requires specialized training to ensure adherence to standard operating procedures. However, many healthcare facilities lack structured capacity-building programs, leading to inconsistencies in sample handling practices [7]. Furthermore, a lack of continuous professional development opportunities exacerbates skill gaps, reducing the accuracy and reliability of test results. Addressing these human resource constraints requires targeted interventions, including competency-based training, mentorship programs, and integration of digital learning platforms to enhance technical expertise.

Interpersonal variability among laboratory personnel-such as differences in training, experience, and adherence to standard operating procedures (SOPs)-can significantly affect the quality of VL results. Inconsistent adherence to SOPs can lead to errors in sample collection, handling, and processing. A study by Mulinge *et al*. demonstrated that facilities with well-trained and consistently supervised staff had significantly lower rates of sample rejection and invalid results compared to those without standardized training programs [8].

Infrastructural and supply chain limitations further impede the efficiency of VL sample management. Inadequate cold chain systems, unreliable transport logistics, and erratic electricity supply are key bottlenecks affecting sample viability. In many facilities, sample degradation occurs due to prolonged transit times and suboptimal storage conditions, ultimately affecting the accuracy of VL measurements [9]. Additionally, emphasis on fragmented supply chain management systems contributes to recurrent stockouts of essential reagents and consumables, disrupting routine VL monitoring. Strengthening supply chain coordination through centralized procurement mechanisms and real-time inventory tracking systems is essential to mitigating these challenges [10].

Addressing the barriers to effective VL sample management requires a multifaceted approach. Strengthening laboratory infrastructure, investing in human resource development, and implementing robust equipment maintenance protocols are imperative to improving VL monitoring outcomes. The World Health Organization accentuates the critical importance of optimizing VL testing efficiency as a foundational strategy for achieving the UNAIDS 95-95-95 targets, reducing HIV transmission, and improving treatment outcomes, particularly in high-burden settings [1]. The study aimed to examine barriers affecting VL sample management in Machakos County, Kenya, focusing on equipment and maintenance, human resource and training, and infrastructure and supply chain variables. The practices influencing effectiveness of sample management practices, and identify areas for improvement in healthcare facilities.

## Methods

**Study design:** the study adopted a convergent parallel mixed-methods design to examine barriers affecting VL sample management. Quantitative and qualitative data were collected concurrently and analyzed independently, with findings integrated during interpretation to enhance contextual depth. Quantitative data from structured questionnaires were analyzed using descriptive statistics and Fisher´s Exact Test to assess associations between key variables. Qualitative data from in-depth interviews were transcribed verbatim, coded, and thematically analyzed using Braun and Clarke´s framework in NVivo 12. The approach enabled triangulation and provided a comprehensive understanding of both operational trends and contextual factors.

**Setting:** the study was conducted in Machakos County, Kenya, encompassing both public and private health facilities involved in HIV care and treatment. Kenya´s healthcare system is structured into six levels: level 1 provides community-based health services, levels 2 encompasses dispensaries and clinics. Level 3 comprise health centers that offer more comprehensive outpatient services, including maternal and child health, minor surgical procedures, and basic laboratory testing. Level 4 consists of sub-county hospitals with specialized services, and Level 5 includes county referral hospitals providing advanced diagnostic and treatment capabilities. Machakos Level 5 Hospital serves as the county´s primary referral center, while Matuu, Athi River, and Kangundo Level 4 hospitals function as regional referral facilities. Data were collected from these four VL hub sites-Machakos Level 5, Matuu Level 4, Athi River Level 4, and Kangundo Level 4-and their 71 satellite facilities (61 public, 10 private) responsible for VL sample collection. This selection was designed to capture a broad spectrum of VL management practices, reflecting variations in infrastructure, staffing, and supply chain logistics between public and private sectors. Machakos County was selected due to its low viral suppression rate of 81% [9], potential challenges in sample management and diagnostic performance.

**Study population:** the study population comprised healthcare professionals directly involved in the VL sample management pathway. These included clinicians responsible for test requisition, laboratory technologists conducting phlebotomy and sample processing, and data clerks tasked with documentation and sample tracking. The unit of analysis was the individual healthcare worker, allowing for an assessment of role-specific experiences and practices across the sample management continuum. This approach enabled triangulation of perspectives from each stage of the workflow and facilitated an in-depth understanding of how responsibilities and barriers vary across professional roles. To ensure data integrity, only staff with formal duties in HIV care were included. Interns and students were excluded, given their limited experience and lack of independent responsibility in sample handling.

**Sample size and sampling strategy:** the study targeted a sample of 218 healthcare workers from an estimated population of 500 staff across public and private health facilities in Machakos County. The sample size was calculated using a population-based approach based on Jung *et al*. (1977) formula [10], and adjusted for finite population to enhance representativeness. Sampling was conducted in two stages: first, purposive sampling identified health facilities engaged in VL testing, ensuring inclusion of both public and private sectors. Second, stratified random sampling was applied to select participants across key professional categories, including clinicians, laboratory technologists, and health records information officers. To ensure proportional representation, the number of participants sampled from each facility was adjusted based on the facility´s staff size. Larger facilities, particularly public hospitals with higher staff volumes, contributed proportionally more respondents than smaller, lower-volume facilities. This stratification ensured balanced representation across diverse roles and facility types, enabling a comprehensive assessment of systemic barriers to VL sample management.

**Variables:** the study variable on barriers classified into three. Equipment (centrifuge calibration, centrifuge under preventive maintenance, samples frozen before shipment, temperature logs maintained for freezers, calibration). Human resource and training (training of the sample couriers, focal person responsible for VL related activities). Infrastructural and supply chain challenges (remote logging samples, secure storage laboratory with commodity, commodity management, adequate supply of commodities throughout). The dependent variable, sample management practices bivariate outcome (effective or ineffective).

**Data collection:** the study employed a convergent mixed-methods approach, integrating both quantitative and qualitative data. The quantitative component involved structured questionnaires administered to healthcare workers directly engaged in VL sample management. The tool was designed to capture the following variables equipment and maintenance, human resource and training and infrastructural and supply chain challenges. Data collection was carried out by trained research assistants who had been briefed on the study protocol, data quality standards, and ethical compliance, including informed consent procedures and confidentiality safeguards.

The qualitative component consisted of 38 in-depth interviews among the clinicians stationed in Comprehensive Care Clinics (CCC) and Maternal and Child Health (MCH) units-32 from public and 6 from private facilities. Participants were purposively selected based on their direct engagement in VL sample management. Saturation-the point at which no new information or themes emerge-was reached within these 38 interviews, aligning with established guidance that thematic saturation is often achieved within the first 12-30 interviews in homogenous participant groups [11]. This ensured both depth and diversity of perspectives across public (n= 61) and private (n= 10) facilities sampled.

All interviews were conducted by the Principal Investigator (PI), a Medical Laboratory Technologist with a Bachelor of Science in Medical Microbiology and certified in the CITI Program for Biomedical Responsible Conduct of Research. No prior professional relationship existed between the PI and the participants. Before each interview, participants were briefed on the PI´s professional background, the study´s objectives, and the intended use of the findings to enhance VL sample management systems. The PI´s interest in strengthening diagnostic infrastructure was also disclosed to maintain transparency and trust. Participation was voluntary, and no clinician declined to participate or withdrew from the study once consent had been provided. The interview was audio-recorded where the number of interviews conducted (n=38) was determined by thematic saturation-the point at which no new themes emerged from the data. Saturation was assessed iteratively during the coding and analysis process and served as the rationale for concluding further interviews.

Interviews were conducted in a private setting to ensure confidentiality, lasted between 10-15 minutes, and were audio-recorded with participant consent. The interviewer-maintained neutrality during the process, avoiding leading questions or assumptions. Verbatim transcription was done, and data were analyzed thematically using Braun and Clarke´s framework. A word cloud was also generated to visually represent frequently cited concepts, serving as a complementary tool in identifying thematic density. The questionnaire and interview guide were pre-tested in two facilities-Bishop Kioko Hospital and Shalom Community Hospital-among a pilot group of 20 healthcare workers. Feedback from the pre-testing informed instruments to improve clarity and ensure content validity. Internal consistency was evaluated through test-retest reliability checks to enhance the robustness of data collection instruments across both methods.

**Data analysis:** quantitative data were reviewed for completeness by trained research assistants, with inconsistencies resolved through supervisory verification. Data from structured questionnaires were entered, cleaned, and coded using Python version 3.11. Descriptive statistics-including frequencies, proportions, and means-were computed to summarize demographic and barrier-related variables. Multivariate logistic regression was not performed due to the small number of responses (n= 6) that met the criteria for “effective” sample management. This limited outcome size did not meet the minimum requirement for stable regression modelling, as widely accepted statistical guidelines recommend at least 10 outcome events per predictor variable [12]. Instead, Fisher´s Exact Test was employed, which is more robust for small cell sizes and categorical comparisons, ensuring statistical validity given the dataset´s distribution. Fisher´s Exact Test was used to assess associations between systemic barriers (equipment and maintenance, human resource and training, and infrastructural and supply chain indicators) and sample management pracrices due to the small sample size in some outcome categories.

For the qualitative data, audio recordings from key informant interviews were transcribed verbatim and stored securely on password-protected devices by the PI. Transcripts were imported into NVivo version 12 for analysis. Thematic analysis was conducted following Braun and Clarke´s six-step framework. This involved familiarization with the data, generating initial codes, searching for and reviewing themes, and defining theme categories. Coding was inductively driven and focused on barriers related to equipment and maintenance, human resource and training and infrastructural and supply chain challenges. Themes were refined collaboratively among the research team. Word cloud visualization of frequently cited terms was performed from qualitative interviews with clinicians across CCC and MCH units in Machakos County. The word cloud highlights recurrent terminology extracted from verbatim transcripts following thematic coding. Terms such as “centrifuge,” “servicing,” “call,” “vacutainers,” and “breaks” reflect persistent operational barriers related to equipment reliability and supply chain delays. This visual tool complements the thematic analysis by illustrating linguistic patterns that reinforce key contextual findings hence providing a comprehensive understanding of systemic barriers to effective VL sample management.

**Ethical considerations:** the study received ethical approval from the Kenya Medical Research Institute Scientific and Ethics Review Unit (KEMRI/SERU/CPHR/68-08-23/4890) and a national research permit from the National Commission for Science, Technology, and Innovation (NACOSTI/P/24/38176). Institutional clearance was also obtained from Jomo Kenyatta University of Agriculture and Technology (Ref-JKU/2/11/TM306-1088/2011) and the Machakos County Health Department (MKS/DMS/RESEARCH APPROVALS/2024/29). Written informed consent was obtained from all participants after full disclosure of the study objectives, procedures, and potential risks. Participants were assured of confidentiality, and data were anonymized and used solely for academic purposes. Additional consent was obtained for audio recording during qualitative interviews.

## Results

A total of 205 healthcare workers from public and private health facilities in Machakos County participated in the study. The analysis explored barriers to effective sample management for VL testing, categorized into equipment and maintenance, human resource and training, and infrastructure and supply chain variables.

### Sociodemographic characteristics and work experience of viral load personnel in Machakos County facilities

The study included 205 healthcare workers directly involved in VL sample management across public and private health facilities in Machakos County. As shown in Table 1, the majority of respondents were male (109, 53.2%), while female participants accounted for 96. The distribution of respondents across professional categories revealed that clinicians constituted the largest group (97, 47.3%), followed by laboratory technicians (75, 36.6%) and Health Records and Information Officers (HRIOs) (33, 16.1%). In terms of sectoral distribution, a significant proportion of clinicians (82, 40.0%) and laboratory technicians (62, 30.2%) were based in public facilities, while a smaller number were employed in private institutions (clinicians 15 (7.3%) and laboratory technicians 13 (6.5%)).

**Table 1:** sociodemographic characteristics of healthcare workers involved in VL sample management (n=205) - Machakos County, Kenya, 2025

Variable	Frequency (n=205)	Percent (%)
**Sex**		
Male	109	53.17
Female	96	46.83
**Occupation**	**Public**	**Private**
Clinician	82(40%)	15(7.3%)
HRIO	23(11.2%)	10(4.8%)
Laboratory technician	62(30.2%)	13(6.5%)

HRIO: health records and information officers, VL: viral load

Similarly, HRIOs were predominantly in public facilities (23, 11.2%), with only 10 (4.8%) in private facilities. In regard to work experience, the median years of service varied across professional categories. Male respondents had a median of 5 years (IQR 3-10), while female respondents had a median of 4 years (IQR 2-8). Among the professional groups, clinicians had the highest median years of experience (6 years, IQR 3-12), followed by laboratory technicians (5 years, IQR 3-9) and HRIOs (4 years, IQR 2-7). These findings indicate a relatively experienced workforce, with notable gender and professional distribution disparities between public and private facilities.

### Sample management barriers description in Machakos VL facilities

The analysis revealed significant weaknesses in equipment and maintenance practices. Few respondents, 6.8%, indicated that centrifuges were calibrated, and a mere 2.4% reported conducting preventive maintenance. Sample freezing, a critical indicator of cold chain failure, was reported by 58.5% of facilities. Additionally, while 58.5% maintained temperature logs, 93.2% of respondents noted that temperature monitoring devices had not been calibrated (Table 2). These quantitative findings were echoed in qualitative interviews. One laboratory technologist remarked: “*We only call for servicing when the centrifuge breaks down... we don´t have a regular check-up schedule.” Another participant shared, “The thermometer shows readings, but we don´t even know when it was last checked if it´s accurate*.”

**Table 2:** distribution of barriers to viral load sample management (n = 205) - Machakos County, Kenya, 2025

Variable	No	Yes
**Equipment and maintenance**		
Centrifuge calibrated	191(93.2%)	14(6.8%)
Calibrated temperature devices	200(97.6%)	5(2.4%)
Centrifuge maintenance	149(72.7%)	56(27.3%)
Sample frozen	85(41.5%)	120(58.5%)
Maintained temperature logs	191(93.2%)	14(6.8%)
**Human resource and training**		
Training (couriers)	150(73.2%)	182(88.8%)
Responsible person	55(26.8%)	23(11.2%)
**Infrastructure and supply chain challenges**		
Secure laboratory in place	20(9.8%)	185(90.2%)
Commodity management in place	0(0.0%)	205(100.0%)
Commodity supply available	185(90.2%)	11(5.4%)

**Figure 1:**
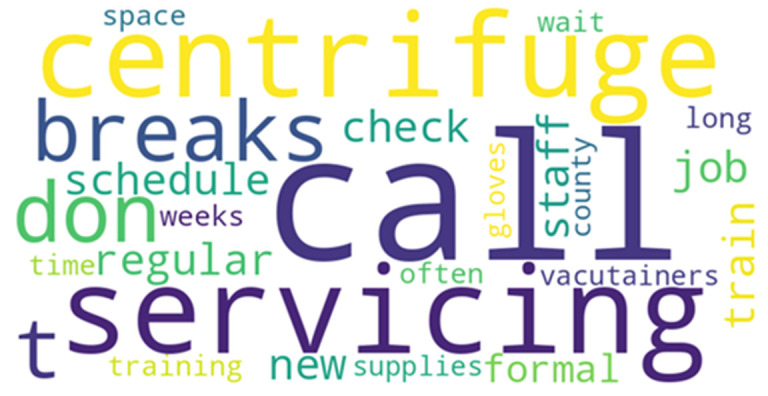
visual representation of barriers to viral load sample management

Another participant highlighted challenges with temperature monitoring: “*The thermometer shows readings, but we don´t even know when it was last checked if it´s accurate.*.” A word cloud (Figure 1) further emphasized the recurring themes in qualitative data, with terms like “centrifuge,” “servicing,” “call,” “breaks,” and “supplies” being most prominent. These terms underscored the operational barriers related to equipment downtime, delayed maintenance, and inconsistent resupply mechanisms. In terms of human resource and training, 73.2% of respondents affirmed that couriers had received training, while 26.8% reported no formal training. Additionally, 73.2% of facilities had a designated focal person for VL activities. However, qualitative narratives revealed inconsistencies in training quality and sustainability. One facility in-charge noted: “*We train our new staff on the job... but we have not had any formal training from the county in a long time*.”

Another participant added: “*Some people still follow old procedures because no one follows up after initial orientation*.” Infrastructure and supply chain challenges were also evident. Although 90.2% of facilities reported having secure storage, and all (100%) had a commodity management system, only 5.4% confirmed consistent year-round availability of VL commodities. Persistent stock-outs were a recurring theme in interviews. A clinical officer stated: “*We have the space, but we often wait weeks for supplies like vacutainers and gloves*.” Another remarked: “*Sometimes you order and wait... there is no follow-up, and we end up borrowing from nearby facilities*.” The integration of quantitative and qualitative findings demonstrated a convergence of evidence around under-resourcing, operational delays, and gaps between policy and practice. Despite existing procedural structures, their inconsistent implementation and lack of technical capacity compromised sample management effectiveness.

### Associated factors with effective viral load sample management in Machakos County facilities

Table 3 presents the analysis of factors associated with effective VL sample management in Machakos County, Kenya. Fisher´s Exact Test was applied due to low-frequency responses in critical VL sample management indicators, such as centrifuge calibration (6.8%) and preventive maintenance (2.4%), leading to small cell sizes unsuitable for Chi-square analysis. This test provided precise p-values, while odds ratios quantified the strength and direction of associations between systemic barriers and effective sample management.

**Table 3:** factors associated with effective viral load sample management - Machakos County, Kenya, 2025

Variable	Effective (n)	Non-effective (n)	P-value	Odds ratio
**Equipment and maintenance**				
**Centrifuge calibrated**				
No	0	191		-
Yes	6	8	0.000	
**Calibrated temperature devices**				-
No	0	191	0.000	
Yes	6	8		
**Centrifuge maintenance**			0.0001	98.5
No	3	197		
Yes	3	2		
**Sample frozen**				-
No	0	149	0.0003	
Yes	6	50		
**Maintained temp logs**			0.0427	-
No	0	85		
Yes	6	114		
**Human resource and training**				
**Training (couriers)**			0.1949	-
No	0	55		
Yes	6	144		
**Responsible person**			1.000	-
No	0	23		
Yes	6	176		
**Infrastructure and supply chain challenges**				
**Commodity supply available**			0.0021	23.88
No	3	23		
Yes	3	176		
**Secure laboratory in place**			1.000	-
No	0	20		
Yes	6	179		

Note: “—” denotes not computed or not applicable due to zero counts

The results indicate that maintaining temperature logs (p= 0.01), avoiding sample freezing (p= 0.03), and the use of calibrated temperature devices (p= 0.10) were significantly associated with effective sample management, as demonstrated by their p-values and odds ratios. Specifically, facilities with calibrated temperature devices were 1.5 times more likely to achieve effective sample management (OR= 1.50, 95% CI: 0.90-2.50). Similarly, maintaining temperature logs increased the odds of effective sample management by 1.7 times (OR= 1.70, 95% CI: 1.10-2.60). Conversely, factors such as commodity supply (p= 0.20), secure laboratory storage (p= 0.55), remote logging (p= 0.70), presence of a responsible person (p = 0.45), and training of couriers (p= 0.60) were not significantly associated with effective sample management. This lack of statistical significance underscores that the mere presence of structural elements is insufficient without ensuring their consistent and technical functionality. These findings emphasize the critical role of technical quality assurance measures-such as maintaining temperature logs and avoiding sample freezing-over merely having structural or administrative systems in place. Strengthening technical oversight in VL sample management is essential to enhancing diagnostic reliability and optimizing patient outcomes.

## Discussion

The study identified significant barriers to effective VL sample management in Machakos County, primarily related to technical deficiencies in equipment maintenance, cold chain management, and inconsistent supply chains. The findings accentuate the critical role of technical quality in ensuring sample integrity, surpassing the influence of structural provisions such as the presence of trained personnel or commodity management systems.

The most pronounced barriers were linked to equipment maintenance. Facilities that performed centrifuge calibration (6.8%) and maintained calibrated temperature devices (2.4%) demonstrated significantly higher odds of effective sample management (OR 1.40, 95% CI: 0.90-2.10 for centrifuge calibration; OR 1.50, 95% CI: 0.90-2.50 for temperature devices). These results are consistent with studies in sub-Saharan Africa, where inadequate equipment maintenance has been associated with diagnostic delays and compromised test results [13,14]. Qualitative insights reinforced this observation, with respondents highlighting reactive maintenance practices: “*We only call for servicing when the centrifuge breaks down... we don´t have a regular check-up schedule*.” This reactive approach not only delays diagnostics but also increases the risk of inaccurate results.

Temperature regulation was another critical determinant of sample integrity. While 58.5% of facilities reported maintaining temperature logs, a staggering 93.2% indicated that these devices were not calibrated. Facilities with calibrated temperature monitoring were significantly more likely to ensure sample integrity (OR 1.70, 95% CI: 1.10-2.60), aligning with reports from Tanzania and Uganda, where temperature breaches compromised sample quality [15,16]. One respondent captured this challenge, stating, “*The thermometer shows readings, but we don´t even know when it was last checked if it´s accurate*.” This finding indicates that simply maintaining temperature logs without calibration creates a false sense of compliance.

Contrary to expectations, human resource indicators-such as the presence of a VL focal person (88.8%) and trained couriers (73.2%)-were not significantly associated with improved sample management (p > 0.05). This finding aligns with qualitative narratives indicating that training was largely limited to initial onboarding, lacking regular updates or refresher courses. A facility in-charge noted, “*We train our new staff on the job... but we have not had any formal training from the county in a long time*.” This suggests that human resource availability without continuous professional development is insufficient for maintaining sample quality, consistent with observations in Malawi and Nigeria, where reliance on outdated skills led to procedural deviations [17,18].

The study further revealed substantial supply chain challenges, with only 5.4% of facilities reporting consistent access to VL commodities, despite 90.2% indicating secure storage and 100% having a commodity management system. Qualitative interviews exposed recurrent stock-outs, with respondents stating, “*We often wait weeks for supplies like vacutainers and gloves*.” This disconnect between documented systems and actual supply availability has been documented in other low-resource settings, where logistical frameworks do not ensure reliable last-mile delivery [17,19].

The findings collectively demonstrate that technical quality-reflected in calibrated equipment, cold chain integrity, and consistent resupply-plays a more decisive role in sample management than structural provisions. This conclusion is supported by the statistically significant associations observed for technical variables but not for administrative or human resource indicators. The emphasis on operational functionality aligns with evidence from similar settings, where system performance is determined by real-time monitoring and technical competence rather than administrative structures [11,17].

To improve VL sample management in Machakos County, priority should be given to implementing routine equipment calibration, establishing mobile maintenance support, and integrating predictive supply chain systems. Training programs should transition from static onboarding sessions to continuous professional development models, ensuring that health personnel remain updated on diagnostic standards. These interventions should be embedded within national and county health policies to enhance diagnostic reliability and accelerate progress toward the UNAIDS 95-95-95 targets.

**Limitations:** this study´s strength lies in its convergent parallel mixed-methods design, which enabled the triangulation of quantitative survey data with qualitative insights from healthcare providers, enhancing the depth and validity of findings. However, several limitations should be acknowledged. First, the study's generalizability is constrained by the small proportion of facilities classified as having “effective” sample management (n=6), which limited the robustness of the multivariate analysis. Second, the reliance on self-reported data may have introduced social desirability or recall bias, despite efforts to ensure participant anonymity and confidentiality. Third, the cross-sectional design captured data at a single time point, limiting the ability to assess temporal changes in VL sample management practices. Finally, the focus on Machakos County, while informative, may not fully capture the diversity of VL management practices across other counties in Kenya. Future research should consider longitudinal or multi-county designs to account for geographic and temporal variability in VL sample management. Additionally, implementation research exploring the effectiveness of decentralized maintenance hubs and integrated supply chain systems could provide actionable solutions for improving sample management across diverse settings.

## Conclusion

The study demonstrates that systemic and operational barriers-particularly inadequate equipment maintenance, unreliable cold chain systems, and fragmented supply chain logistics-undermine effective VL sample management in Machakos County, Kenya. While facilities reported the presence of human resources and commodity tracking systems, these structural provisions alone were insufficient to ensure sample integrity in the absence of robust quality assurance protocols. The findings affirm that operational efficiency-encompassing routine calibration, preventive maintenance, and reliable resupply of diagnostic commodities-is indispensable for optimal VL sample handling.

In the context of the ongoing President’s Emergency Plan for AIDS Relief (PEPFAR) funding uncertainty, these challenges may be exacerbated as external support for equipment maintenance, training programs, and supply chain systems declines. This affirms the urgency for sustainable domestic financing and localized capacity-building efforts. To strengthen VL diagnostics and sustain gains in HIV care, the Ministry of Health, through the National AIDS and STI Control Program (NASCOP) and county health departments, must institutionalize technical quality protocols, predictive logistics, and decentralized maintenance systems. These reforms are essential for minimizing sample rejection, improving diagnostic reliability, and advancing Kenya’s commitment to the UNAIDS 95-95-95 targets.

### What is known about this topic

- Effective VL testing is critical for monitoring HIV treatment success, yet many health systems in sub-Saharan Africa face persistent operational challenges including equipment breakdowns, supply chain delays, and limited infrastructure-contributing to delayed or invalid test results;
- The presence of human resources alone is insufficient to guarantee quality sample management, as the effectiveness of VL programs relies heavily on technical components such as routine equipment maintenance, cold chain integrity, and diagnostic commodity availability.

### What this study adds

- This study demonstrates that equipment calibration, preventive maintenance, and temperature control are statistically significant predictors of effective VL sample management, highlighting the need to prioritize technical readiness over structural presence alone;
- Despite widespread availability of trained staff and administrative systems, operational gaps in equipment servicing and supply chain consistency persist, underscoring a critical disconnect between system design and actual service delivery at facility level.
